# Structure of Salvioccidentalin, a Diterpenoid with a Rearranged *neo*-Clerodane Skeleton from *Salvia occidentalis*

**DOI:** 10.3390/molecules16119109

**Published:** 2011-10-31

**Authors:** Miguel Ángel Jaime-Vasconcelos, Bernardo Antonio Frontana-Uribe, José Antonio Morales-Serna, Manuel Salmón, Jorge Cárdenas

**Affiliations:** Instituto de Química, Universidad Nacional Autónoma de México, Circuito Exterior, Ciudad Universitaria, 04510 México D. F., México; Email: mguelajv@gmail.com (M.A.J.-V.); bafrontu@unam.mx (B.A.F.-U.); morser@unam.mx (J.A.M.-S.); salmon@unam.mx (M.S.)

**Keywords:** *Salvia occidentalis*, Labiatae, diterpene, *neo*-clerodane, NMR

## Abstract

From the aerial parts of *Salvia occidentalis* (Labiatae) a new diterpenoid with a rearranged *neo*-clerodane skeleton was isolated. This new compound was named salvioccidentalin and its structure was established by spectroscopic means. A probable biogenetic relationship with salvigenolide from *S. fulgens* and salvileucalin A and spiroleucantholide from *Salvia leucantha* is proposed.

## 1. Introduction

The Labiatae family comprises about 220 genera and 4,000 species. Some species of this family have economic importance due to their essential oil content [1] or their use in folk medicine, which is frequently related to the diterpenoid content of the plants [2,3,4]. One of the largest genus of the family, *Salvia* L., is represented by over 900 species, and is widely distributed in various regions of the world [5]. In Mexico, the Labiatae family is well represented, mainly by the genus *Salvia*, subgenus Calosphace, which is one of the largest in our country with over 257 species [6]. The diterpenoids isolated from Mexican *Salvia* species mainly have a clerodane skeleton or a rearranged skeleton biogenetically related to a clerodanic precursor [7,8].

As part of our continuing systematic study of the genus *Salvia *[5], in this paper we describe the structure and stereochemistry of salvioccidentalin (**1**), a new diterpenoid with a novel rearranged *neo*-clerodane skeleton, isolated from the aerial parts of *Salvia occidentalis* Sw. (Subgenus Calosphace, section Microsphace Benth) [9], a shrub which grows in the state of Jalisco, Mexico.

## 2. Results and Discussion

Compound **1** was isolated as white solid, mp 165–168 °C, and showed a molecular formula C_20_H_20_O_5_ by mass spectrometry. Its IR spectrum exhibited the characteristic absorptions for double bonds (1,673 cm^−1^) and γ-lactone functions (1,766 cm^−1^).

**Figure 1:**
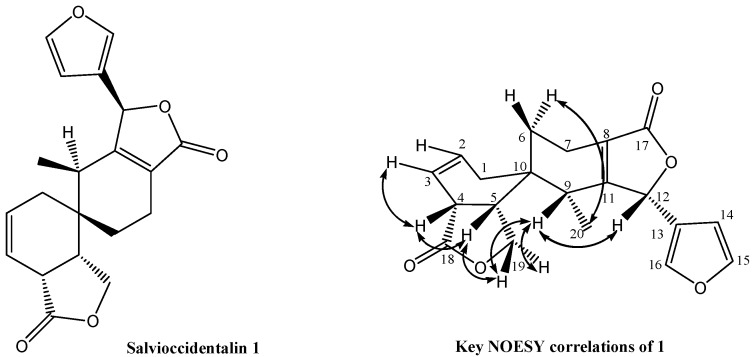
Salvioccidentalin (**1**).

The ^1^H- and ^13^C-NMR spectra (Table 1) helped to establish compound **1** as a rearranged clerodane diterpenoid closely related to spiroleucantholide, previously reported from *Salvia leucantha* [10,11]. The ^13^C-NMR spectrum (Table 1) exhibits signals of 20 carbon atoms, including one methyl, four methylenes, nine methines and six quaternary carbons, which were assigned with the aid of DEPT, HSQC and HMBC. The signals at δ 175.9 (C-18) and 172.1 (C-17) correspond to γ-lactone carbonyl groups, and the signals at δ 77.6 (C-12) and 67.9 (C-19) are characteristic of carbon atoms bearing oxygen. The signals at δ 127.8 (C-2), 119.9 (C-3), 126.2 (C-8) and 163.9 (C-11) were ascribed to two double bonds. The ^1^H-NMR spectrum showed signals for a secondary methyl group at δ 0.74 (d, *J* = 7.5 Hz, H-20), the β-substituted furan protons gave characteristic signals at δ 6.23 (1H, dd, *J* = 1.5, 0.5 Hz, H-14), 7.45 (1H, t, *J* = 1.5 Hz, H-15) and 7.58 (1H, br s, H-16). A broad singlet at δ 5.68 was attributed to C-12 allylic proton bound to a carbon bearing oxygen. The fragment at m/*z* 95 in the mass spectrum of **1** (see EIMS) supports the existence of the β-substituted fu ran ring with an oxygenated function at C-12. The signals due to vinylic protons were observed at δ 5.93 (1H, dddd, *J* = 10.5, 6.0, 2.5, 2.5 Hz) and 5.69 (1H, br d, *J* = 10.5 Hz), which were ascribed to H-2 and H-3, respectively. The ^1^H-^1^H COSY spectrum reveals connectivity from C-6 to C-7 methylene protons, showing the signals at δ 1.6 (ddd, *J* = 14.5, 12.0, 6.5 Hz, H-6_ax_), 1.85 ( dd, *J* = 14.5, 6.5 Hz, H-6_eq_), 2.25 (m, H-7_ax_) and 2.47 (dd, *J* = 19.0, 6.5 Hz, H-7_eq_). Double doublets at δ 4.21 (H-19 *pro-S*, *J* = 8.5, 8.5 Hz) and 4.05 (H-19 *pro-R*, *J* = 11.0, 8.5 Hz) were assigned to C-19 methylene protons. From NOESY correlation between 1_ax_ and 19A it was possible assigned the stereodescriptors *pro-S* and *pro-R*. The multiplet at δ 3.14 was assigned to H-4 according to HMBC coupling between H-4 and C-18 (see Table 1). Analyses of the same spectra and of the signals observed in the 2D experiments (COSY and HSQC), led to the assignment of the double doublet of doublets found at δ 2.75 (*J* = 11.0, 8.5, 8.5 Hz) to the C-5 proton. The relative configuration of **1** was assigned on the basis of ^1^H-^1^H vicinal coupling constant analysis and NOESY experiments.

**Table 1:** NMR spectroscopic data for compound **1** (^1^H: 500 MHz, ^13^C: 125 MHz; CDCl_3_).

Position	δH (mult, *J* in Hz)	COSY (H-H)	NOESY (H-H)	δC (DEPT)	HMBC (H→C)
1 H_eq_	1.92 (br dd, *J* = 18.0, 4.0)	H-1_ax_, H-2			2, 3, 5, 6, 9, 10
1 H_ax_	2.10 (br dddd, *J* = 18.0, 2.5, 2.5, 2.5)	H-1_eq_, H-3, H-4	H-19A	31.2 (CH_2_)	
2	5.93 (dddd, *J* = 10.5, 6.0, 2.5, 2.5)	H-1_eq_, H-3	H-1_eq_, H-3	127.8 (CH)	4, 10
3	5.69 (br d, *J* = 10.5)	H-1_eq_, H-2, H-4	H-2, H-4	119.9 (CH)	1, 4, 5
4	3.14 (m)	H-1_eq_, H-3, H-5	H-3, H-5, H-6_eq_	40.8 (CH)	3, 18, 19
5	2.75 (ddd, *J* = 11.0, 8.5, 8.5)	H-4, H-19A, H-19B	H-4, H-19B	37.4 (CH)	1, 3, 4, 19
6 H_ax_	1.60 (H_ax_, ddd, *J* = 14.5, 12.0, 6.5)	H-6_eq_, H-7_ax_, H-7_eq_	H-6_eq_, H-20	24.4 (CH_2_)	7, 8, 9
6 H_eq_	1.85 (H_eq_, dd, *J* = 14.5, 6.5)	H-6_ax_, H-7_ax_	H-6_ax_, H-4		
7 H_eq_	2.47 (H_eq_, dd, *J* = 19.0, 6.5)	H-7_ax_ , H-6_ax_	H-7_ax_	17.5 (CH_2_)	6, 8, 10, 11
7 H_ax_	2.25 (H_ax_, m)	H-7_eq_, H-6_ax_, H-6_eq_	H-7_eq_		
8				126.2 (C)	
9	2.01 (q, *J* = 7.5)	H-20	H-12, H19A, H19B, H-20	35.9 (CH)	5, 6, 8, 10, 11, 12, 20
10				34.3 (C)	
11				163.9 (C)	
12	5.68 (br s)	H-7_ax_, H-7_eq_	H-9, H-16	77.6 (CH)	8, 13, 14, 16
13				120.8 (C)	
14	6.23 (dd, *J* = 1.5, 0.5)	H-15	H-15	108.3 (CH)	13, 15, 16
15	7.45 (dd, *J* = 1.5, 1.5)	H-14, H-16	H-14	144.5 (CH)	13, 14
16	7.58 (br s)	H-15	H-12	141.3 (CH)	13, 15
17				172.1 (C)	
18				175.9 (C)	
19A H *pro-S*	4.05 (dd, *J* = 11.0, 8.5)	H-5, H-19B	H-1_ax_, H-9, H-19B	67.9 (CH_2_)	4, 5, 10, 18
19B H *pro-R*	4.21 (dd, *J* = 8.5, 8.5)	H-5, H-19A	H-5, H-9, H-19A		
20	0.74 (d, *J* = 7.5 Hz)	H-9	H-6_ax_, H-9	14.3 (CH_3_)	9, 10, 11

In ^1^H-NMR spectrum H-4 and H-5 exhibit a coupling constant of 8.5 Hz (gauche) and had a *syn*-periplanar relationship. In the NOESY spectrum, spatial interactions of H-4 with H-5 were observed. This suggests that both protons adopt pseudo-equatorial positions on ring **A**. These analyses permitted the assignment of a *cis*-fused junction for the **A** ring and the lactone ring. In the NOESY spectrum, the C-9 proton at δ 2.01 (1H, q, *J* = 7.5 Hz) shows spectra cross-peaks with the proton at C-12 and both protons at C-19, which indicates that H-9 and H-12 are pseudo-equatorial on the ring **B** and α,β-unsaturated lactone rings respectively. The presence of H-5 and the absence of a proton at C-10, frequently observed in the ^1^H-NMR spectra of clerodane type diterpenoids, combined with HMBC correlations of H-2, H-7_eq_, H-19 *pro-S* and H-20 with C-10 support the idea that C-10 is a quaternary carbon product of a rearrangement on the ring **B** and suggest a spiro system. Finally, the HMBC correlations between the hydrogen H-12 and C-11 and C-13 showed that the furan unit was attached to C-12. These observations are in agreement with those described by Takeya and co-workers for salvileucalin A [10].

Other relevant signals in the ^13^C-NMR spectrum were those at δ 120.8 (C-13), 108.3 (C-14), 144.5 (C-15) and 141.3 (C-16), which confirmed the presence of a β-substituted furan ring. The signal at δ 14.3 was ascribed to the C-20 methyl group. The rest of the signals were assigned to methylenes and methines; thus it is possible to observe signals at δ 31.2 (C-1), 24.4 (C-6), 17.5 (C-7), 40.8 (C-4), 37.4 (C-5) and 35.9 (C-9). The ^13^C-NMR spectra of clerodane type diterpenoids show two sp^3^ quaternary carbons due to C-5 and C-9, however in this case, salvioccidentalin (**1**) showed only one sp^3^ quaternary carbon at δ 34.3, which was assigned to C-10. This fact and the analysis of the data above discussed, all suggest that salvioccidentalin (**1**) possesses a rearranged clerodane skeleton.

**Scheme 1:**
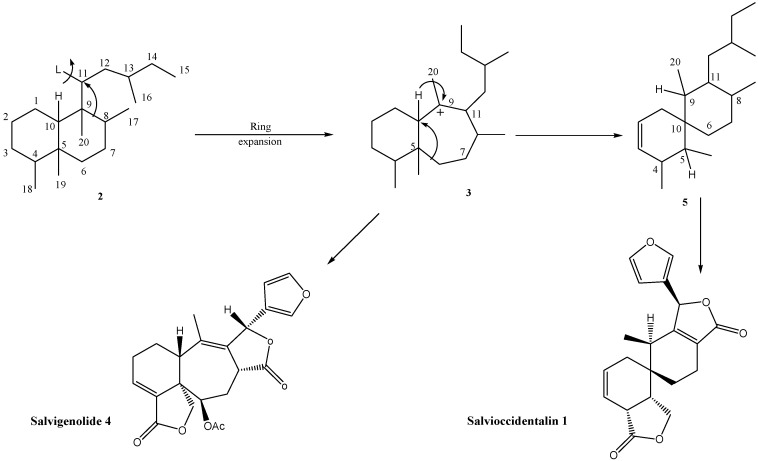
Proposed biogenetic pathway for salvioccidentalin **1**.

**Figure 2:**
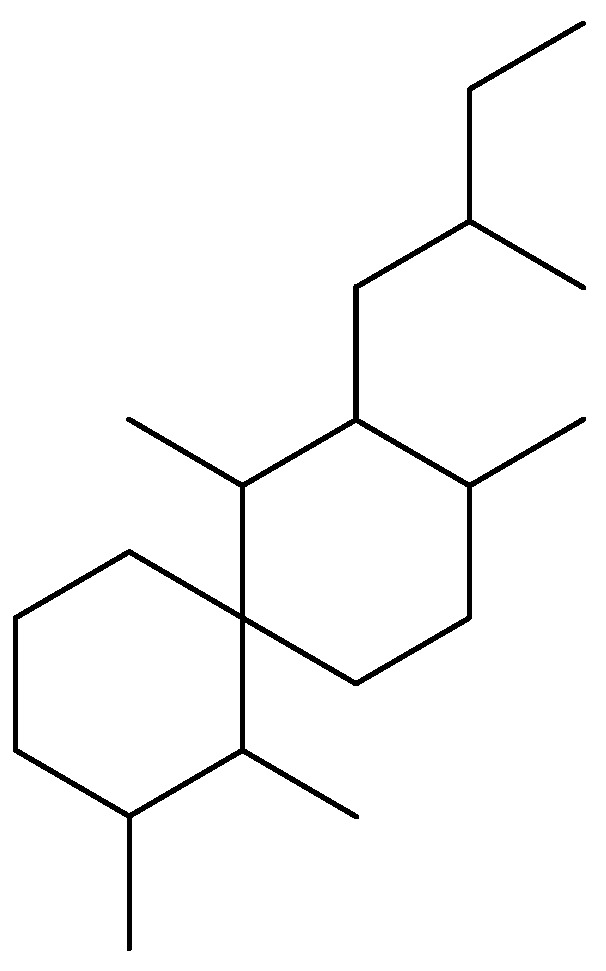
Salvileucalane skeleton.

Salvioccidentalin (**1**) can be biogenetically derived from an intermediate **3** previously proposed as precursor of salvigenolide (**4)**, a rearranged *neo*-clerodane skeleton from *Salvia fulgens* [12]. A hydride migration, followed by 6→10 ring contraction, 1,3-sigmatropic hydrogen shift and concomitant proton loss at C-2 may furnish the rearranged *neo*-clerodane skeleton as outlined in Scheme 1. Additionally, it is noteworthy that the structure of salvioccidentalin (**1**) is very similar to the structure of salvileucalin A [10] and spiroleucantholide [11], which were isolated from *Salvia leucantha*. This entire group of compounds is made of up of rearranged *neo*-clerodanes with an **A**/**B** spiro system, thus we propose a new *neo*-clerodane skeleton named salvileucalane (Figure 2).

## 3. Experimental

### 3.1. General

Infrared spectra were recorded as KBr pellets on a Nicolet FT-IR 750 spectrometer. Mass spectra were recorded with a Jeol JMS-AX505 and Jeol JMS-102A high-resolution mass spectrometer. NMR experiments were conducted on a Varian Unity Plus 500 MHz instrument using CDCl_3_ as solvent, with chemical shifts (δ) referenced to internal standards CHCl_3_ (7.26 ppm ^1^H, 77.0 ppm ^13^C) or TMS as an internal reference (0.00 ppm). Chemical shifts are in parts per million (ppm). For HMBC experiments the delay (1/2*J*) was 70 ms and for the NOESY experiments the mixing time was 150 ms. Column chromatography was performed using silica gel GF_254_ and flash chromatography silica gel (230–400 mesh) and employed a solvent polarity correlated with TLC mobility. Developed TLC plates were visualized under a short-wave UV lamp and by heating plates that were dipped in Ce(SO_4_)_2_.

### 3.2. Extraction and Isolation

The plant *Salvia occidentalis* was collected in Santa Anita, in the State of Jalisco, Mexico, in June 2000 and identified by Dr. Irene Díaz from the Instituto de Biología, UNAM (col:12, MEXU: 967715). Dried and powdered aerial parts (893 g) were extracted with acetone (4 Lt) for five days at room temperature. The solvent was removed *in vacuo* and the residue was subjected to liquid-liquid extraction using a mixture of hexane-benzene (100 mL, 1:1) and methanol-water (100 mL, 3:1). This procedure allowed to obtain a non polar phase and a polar phase and then, the methanol was eliminated under reduced pressure from the polar (methanol-water) phase. The aqueous residue was subjected to extractions with EtOAc (3 × 100 mL). The combined EtOAc layers were dried over Na_2_SO_4_, filtered and concentrated under vacuum to give 4.4 g of residue, which was purified by column chromatography eluted with *n*-hexane/EtOAc (100:0 to 0:100) to afford 11 fractions (1–11). Extensive purification by column chromatography of the fraction 5 (eluted with *n*-hexane/EtOAc 60:40) led to the isolation of the salvioccidentalin (**1**, 10 mg) as a white solid, mp 165–168 °C; [α]_D_ +36.4° (*c *0.10, CHCl_3_). ^1^H-NMR (500 MHz, CDCl_3_) and ^13^C-NMR (125 MHz, CDCl_3_), see Table 1. IR (KBr): 2927, 2855, 1766, 1673, 1440, 1184, 1128, 1026, 1007, 929 cm^−1^. EIMS *m/z* (rel. int.): 340 (100), 325 (6.9), 312 (7.5), 295 (8.9), 191 (15), 189 (17.5), 162 (13.1), 145 (10), 129 (8.75), 105 (17.5), 95 (28.125), 91 (33.75), 79 (12.5), 77 (12.5), 44 (8.1), 39 (7.8), 29 (2.5), 28 (8.1). HRMS (FAB: M+1) calcd. for C_20_H_20_O_5_ 341.1389, found 341.1400.

## 4. Conclusions

In summary, a novel diterpene with a rearranged *neo*-clerodane skeleton, named salvioccidentalin, has been isolated from the aerial parts of *Salvia occidentalis* (Labiatae). Additionally, we propose a biogenetic relationship with salvigenolide from *Salvia fulgens*, and salvileucalin A and spiroleucantholide from *Salvia leucantha*, which provides a chemical basis for the botanical relationship established between the section Microsphace and Fulgentes.
